# A Causality between Thyroid Function and Bone Mineral Density in Childhood: Abnormal Thyrotropin May Be Another Pediatric Predictor of Bone Fragility

**DOI:** 10.3390/metabo13030372

**Published:** 2023-03-02

**Authors:** Dongjin Lee, Moon Bae Ahn

**Affiliations:** Department of Pediatrics, College of Medicine, Catholic University of Korea, Seoul 06591, Republic of Korea

**Keywords:** thyroid hormones, bone mineral density, osteoporosis, thyrotropin

## Abstract

Low bone mass can occur in children and adolescents with numerous chronic conditions; however, the influence of abnormal thyroid hormone and thyroid-stimulating hormone (TSH) levels on low bone mineral density (BMD) in children and adolescents remains controversial. Investigating the effects of excessive or deficient thyroid hormone and TSH levels on the risk of childhood bone fragility may provide a better understanding of the role of thyroid function on bone density in the pediatric population. The triiodothyronine (T3), thyroxine (T4), and TSH levels and BMD of 619 children diagnosed with various underlying conditions and whose treatment was completed were simultaneously assessed. The T3, free thyroxine (FT4), and TSH levels were subcategorized based on the age-matched reference range, and the lumbar spine BMD (LSBMD) data were compared. The mean LSBMD z-score was 0.49 ± 1.28, while T3, FT4, and TSH levels were 1.25 ± 0.29 ng/mL, 1.28 ± 0.19 ng/dL, and 2.76 ± 1.87 µU/mL, respectively. Both lumbar and femoral BMD z-scores were lower in children with abnormal TSH levels. TSH abnormality was the strongest risk factor for decreased LSBMD z-scores, and thus could be an early indicator of low BMD in children and adolescents with various underlying conditions.

## 1. Introduction

Thyroid hormone (TH), whose synthesis and secretion are mainly regulated by thyrotropin (a thyroid-stimulating hormone (TSH)), which is secreted from the anterior pituitary lobe, plays a critical role in the metabolism, growth, and development of the human body. Although TH is responsible for the normal growth and differentiation of various organs, bone is one of the major endocrine organs affected by the regulation of TH. Upon the release of thyrotropin, the thyroid gland produces thyroxine (T4) and triiodothyronine (T3), and the effect of TH on the bone occurs when T3 is bound to the α1 and β1 subtypes of nuclear TH receptors, which are mainly expressed in the bone [1]. TH maintains the skeletal homeostasis by differentiating and regulating chondrocytes, osteoblasts, and osteoclasts via the interaction of multiple signaling cascades that affect the bone turnover [2]. The TSH action on the bone remains controversial due to its inhibitory effect on osteoblastogenesis and osteoclastogenesis; therefore, understanding its direct interaction with the bone requires further investigation of the association between TSH receptor activation and skeletal signaling pathways of growth factors such as Indian hedgehog, wingless/integrated, insulin-like growth factor 1, and bone morphogenetic protein [3].

Low bone mass or osteoporosis can occur in children and adolescents with numerous chronic conditions owing to disease progression or treatment-associated factors [4,5,6,7]. Among various causes, endocrine origins such as hypogonadism, Cushing’s syndrome, growth hormone deficiency, hyperparathyroidism, and thyroid disorders are major triggering conditions resulting in low bone mineral density (BMD) and secondary osteoporosis [8]. Both hyperthyroidism and hypothyroidism may lead to the loss of BMD by affecting the bone turnover via the dysregulation of endochondral bone formation and osteoclastogenesis [9]. Excessive levels of TH exert a direct effect on the bone resorption via the cytokine- and cycle adenosine monophosphate-mediated mechanisms, as well as the increased sensitivity of β-adrenergic receptors and bone cells to catecholamines and parathyroid hormone, respectively [10]. On the contrary, TH deficiency not only causes growth retardation and persistent short stature, but also leads to decreased bone turnover due to the disturbance in endochondral ossification induced by a slowed bone formation and resorption process [11].

The influence of abnormal TH and TSH levels, under subclinical or overt conditions, on low BMD in children and adolescents remains controversial. Although the TSH receptor expression in chondrocytes, osteoblasts, and osteoclasts indicates the direct action of TSH on the cartilage and bone, the interpretation remains unclear since the TSH exerts stimulatory and inhibitory effects on osteoblastogenesis and osteoclastogenesis mediated by tumor necrosis factor-α, receptor activator or nuclear factor κ-B ligand, and osteoprotegerin [12]. Proliferating chondrocytes, bone marrow stromal cells, and osteoblasts are the major sites of TH receptor expression. TH is essential for the coordinated progression of endochondral ossification and cartilage matrix mineralization; however, the underlying mechanism of TH’s interaction with the bone matrix needs to be determined [12,13]. In addition, pediatric reference intervals for TH and TSH differ from the adult range and vary with age during childhood; therefore, the accurate identification of abnormal thyroid function is recommended.

Likewise, the thyroid regulation of bone growth and development seems clear, yet the underlying mechanism associated with the action of abnormal thyroid function on the density of a growing skeleton requires further investigation. We hypothesize that abnormal TH or TSH levels affected by various pediatric chronic conditions are associated with low BMD and lead to a higher risk of osteoporosis. Investigating the effects of excessive or deficient T3, T4, and TSH levels on childhood BMD may provide a better understanding of the role of thyroid function in bone density in the pediatric population. Therefore, this study aimed to examine the association between thyroid function and BMD in children with various underlying conditions and to elucidate the association between abnormal thyroid function and low bone mass in order to support the role of early screening of thyroid function in the prevention of osteoporosis.

## 2. Methods

### 2.1. Participants

The present study retrospectively and cross-sectionally reviewed 619 children and adolescents aged 10–18 years who were previously diagnosed with hemato-oncologic, rheumatoid, gastrointestinal, and endocrinologic diseases at a single tertiary care center and completed a disease-associated therapy within at least 1 year prior to the study enrollment. Patients diagnosed with anorexia nervosa, epilepsy, hydrocephalus, or nephrotic syndrome and who were not on any ongoing therapeutic intervention that could affect the skeletal or thyroid function were classified under the miscellaneous category. The serum TH and TSH levels of all patients were simultaneously assessed by performing lumbar and femoral BMD measurements. This study was approved by the Institutional Review Board of the Catholic University of Korea (KC22RISI0387) and was conducted in accordance with the principles of the Declaration of Helsinki. The requirement for informed patient consent was waived as no interventions or further examinations were performed.

### 2.2. Disease Category

#### 2.2.1. Hemato-Oncologic Disease

Patients previously diagnosed with leukemia, lymphoma, histiocytosis, solid organ tumor, or bone marrow failure based on the bone marrow biopsy or radiologic findings underwent surgery, repeat blood transfusions, multidrug chemotherapy, or focal or total body irradiation, with or without peripheral blood stem cell transplantation. The treatment plan was implemented in accordance with the uniform institution-based protocol of the Division of Pediatric Hemato-Oncology.

#### 2.2.2. Rheumatoid Disease

Patients previously diagnosed with systemic lupus erythematosus, juvenile rheumatoid or idiopathic arthritis, Sjogren–Larsson syndrome, Behçet’s disease, and juvenile dermatomyositis, characterized by the presence of disease-specific antibiotics, were treated with glucocorticoids, nonsteroidal anti-inflammatory drugs, and disease-modifying antirheumatic drugs. The treatment plan was implemented in accordance with the uniform institution-based protocol of the Division of Pediatric Rheumatology.

#### 2.2.3. Gastrointestinal Disease

Patients previously diagnosed with inflammatory bowel disease, including Crohn’s disease and ulcerative colitis, based on the gastroduodenocolonoscopic and radiologic findings, were treated with glucocorticoids, 5-aminosalicylic acids, and immune modulators (methotrexate or azathioprine), with or without biologics (such as antitumor necrosis factor or anti-integrin). The treatment plan was implemented in accordance with the uniform institution-based protocol of the Division of Pediatric Gastroenterology and Nutrition.

#### 2.2.4. Endocrine Disease

Patients previously diagnosed with growth hormone deficiency, idiopathic short stature, constitutional delay in growth and puberty, hypophosphatemic rickets, idiopathic osteoporosis, or congenital adrenal hyperplasia underwent hormone replacements. None of the patients with endocrine diseases were previously diagnosed with hyperthyroidism or hypothyroidism or had any history of taking antithyroid drugs or TH therapy. The treatment plan was implemented in accordance with the uniform institution-based protocol of the Division of Pediatric Endocrinology.

### 2.3. Data Collection

#### 2.3.1. Anthropometric Measurement

Height (cm) was measured using a Harpenden Stadiometer (Holtain^®^, Crymych, UK), while weight (kg) was measured using a Simple Weighing Scale (CAS^®^, Seoul, Republic of Korea). The body mass index (BMI) (kg/m^2^) was calculated and converted to age- and sex-matched standard deviation (z) scores, based on the national growth chart [14].

#### 2.3.2. BMD Assessment

The BMD of the lumbar spine (LSBMD), right femur (RFBMD), and left femur (LFBMD) was measured in the anterior–posterior direction using dual-energy X-ray absorptiometry (DXA) (HorizonW DXA system^®^, Hologic Corp., Marlborough, MA, USA), whereas the age- and sex-matched z-scores for areal BMD (g/cm^2^) were determined based on native Normative Pediatric reference data [15]. All DXA measurements were performed by a single radiographer who was blinded to the patients’ clinical history. A low bone mass was defined as an LSBMD z-score of less than 0 to −3.0.

#### 2.3.3. Thyroid Function Test

The T3, FT4, and TSH levels were determined using the blood sample obtained on the same morning that the BMD was measured. The serum T3, FT4, and TSH levels were measured by performing a direct chemiluminescent immunoassay (Atellica IM, Siemens Healthcare Diagnostics Inc.^®^, Malvern, PA, USA) with inter-assay coefficients of variation of less than 13%, 8%, and 10% for T3, FT4, and TSH, respectively. Subsequently, the T3, FT4, and TSH levels were subcategorized into either the high/normal/low or normal/abnormal based on the age-matched reference range (0.83–2.13 ng/mL for T3, 0.8–2 ng/dL for FT4, and 0.6–8 and 0.6–6 µU/mL for TSH of individuals aged 10–15 years and 16–18 years, respectively), and the LSBMD z-scores of each group were compared [16].

### 2.4. Statistical Analysis

For all descriptive variables, the normality of distribution was determined using the Shapiro–Wilk test. Comparison of BMD z-scores between the two (normal/abnormal T3, FT4, or TSH) groups was carried out using the Mann–Whitney U test, while the comparison of BMD z-scores among the three (low/normal/high T3, FT4, or TSH) groups was performed using one-way analysis of variance. The Pearson’s correlation coefficient (r) was generated to represent the linear correlation of LSBMD z-scores with T3, FT4, and TSH levels. Univariate and multivariate regression analyses were then performed to estimate the beta coefficients (β) for factors associated with the LSBMD z-score. Subsequently, a multiple logistic regression analysis of the abnormal T3, FT4, and TSH levels with decreased LSBMD z-scores as dependent variables was performed to determine the odds ratio (OR) and 95% confidence interval (CI) after adjusting for age, sex, BMI, and underlying conditions. All statistical analyses were performed using SPSS software (version 24.0; IBM Corp.^®^, Armonk, NY, USA).

## 3. Results

### 3.1. Demographic and Clinical Characteristics

The clinical characteristics of the 619 children and adolescents are presented in Table 1. Half of the population were male, with a mean age and a BMI z-score of 13.22 ± 3.23 years and 0.03 ± 1.52, respectively. Hemato-oncologic patients accounted for 79.6% of the total study population, with leukemia either originating from the lymphoid or myeloid white blood cells (66.1%) being most prevalent, followed by primary bone marrow failure, including aplastic anemia (9.7%). The most commonly diagnosed rheumatoid diseases were systemic lupus erythematosus (65.6%) and juvenile rheumatoid or idiopathic arthritis (12.5%). The overall proportions of patients with gastrointestinal (3.7%) and endocrinological disorders (3.1%) were similar. The miscellaneous conditions included anorexia nervosa (13), epilepsy (3), hydrocephalus (2), and nephrotic syndrome (2). The ages at diagnosis of an underlying disease were 8.67 ± 13.03, 12.29 ± 3.75, 13.59 ± 2.48, 9.48 ± 4.75, and 9.01 ± 5.95 years for the hemato-oncologic, rheumatoid, gastrointestinal, endocrinologic, and miscellaneous groups, respectively. The mean LSBMD z-score was 0.49 ± 1.28. Approximately 37.7% of the patients had a mean LSBMD z-score of less than −1.0. Meanwhile, the femoral BMD z-scores were lower than the LSBMD z-scores. More than 90% of all subjects showed normal thyroid function on DXA assessment, and those who initially showed abnormal T3, FT4, or TSH levels did not undergo TH replacement or antithyroid drug therapy, as the follow-up concentrations returned to normal within 6 months.

### 3.2. LSBMD and Thyroid Function of Patients with an Underlying Condition

**Figure 1:**
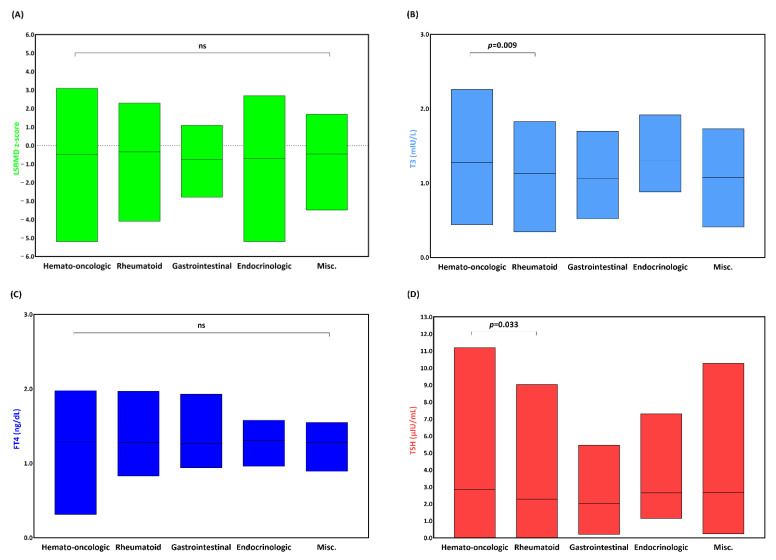
Floating bars comparing (**A**) lumbar spine bone mineral density (LSBMD), (**B**) triiodothyronine (T3), (**C**) free thyroxine (FT4), and (**D**) thyrotropin (TSH) based on underlying conditions. Each end of the bars represents minimum to maximum values, while a line drawn indicates a mean value. The *p*-value is listed when it is significant, otherwise it is noted as non-specific (ns).

LSBMD and thyroid function were compared based on the five underlying conditions (Figure 1). The magnitude of the LSBMD z-scores for hemato-oncologic and endocrinologic diseases was the highest (the highest and lowest LSBMD z-scores were 3.1 and −5.2 for hemato-oncologic patients and 2.7 and −5.2 for endocrinologic patients, respectively). Meanwhile, the mean LSBMD z-scores did not show a significant difference among the study groups. The serum T3 and TSH levels were higher in the hemato-oncologic group compared with that in the rheumatoid group (1.28 ± 0.27 mIU/L vs. 1.13 ± 0.32 mIU/L, *p* = 0.009; 2.86 ± 1.87 µIU/mL vs. 2.29 ± 1.65 µIU/mL, *p* = 0.033); however, no significant difference was observed between the rest of the study groups. In contrast, no significant difference was observed in the serum FT4 level in all study groups.

### 3.3. BMD Status under Abnormal Thyroid Function

**Figure 2:**
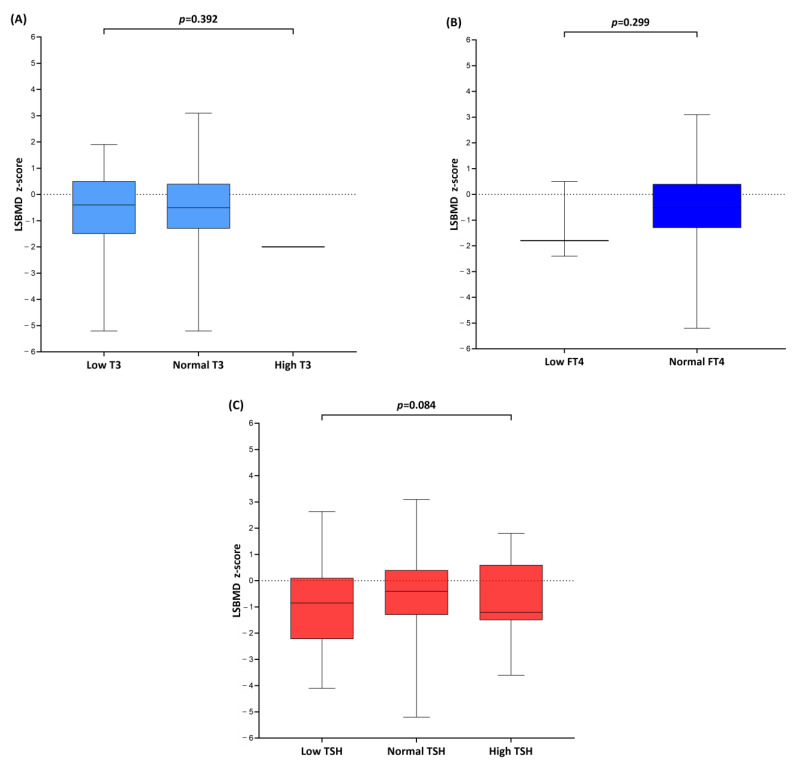
Box-and-whisker plots were drawn to compare lumbar spine bone mineral density (LSBMD) z-scores when (**A**) triiodothyronine (T3), (**B**) free thyroxine (FT4), and (**C**) thyrotropin (TSH) levels were low, normal, or high. The lines inside the boxes indicate the mean value while the whiskers represent the lowest and highest observations, respectively. * No subjects with high FT4.

The serum T3, FT4, and TSH levels were subcategorized into either normal/abnormal (Table 2) or high/normal/low (Figure 2) based on the age-matched reference range, and the BMD z-scores were compared. Both lumbar (*p* = 0.018) and femoral (*p* = 0.036 for left, *p* = 0.007 for right) BMD z-scores were significantly lower in the abnormal TSH group compared with that in the normal TSH group; meanwhile, the left femoral BMD z-scores were the lowest, while the z-score difference was greatest in patients with abnormal serum TSH levels compared with that in patients with normal serum TSH levels. In contrast, no significant difference in BMD z-scores was observed between patients with abnormal and normal T3 or FT4 levels (Table 2).

Figure 2 demonstrated the corresponding LSBMD z-scores for low, normal, or high serum T3, FT4, and TSH levels. The LSBMD z-score difference was not significant when the serum T3, FT4, or TSH levels were either low or high. None of the participants had hyperthyroxemia.

### 3.4. Causal Association between BMD and Thyroid Function

**Figure 3:**
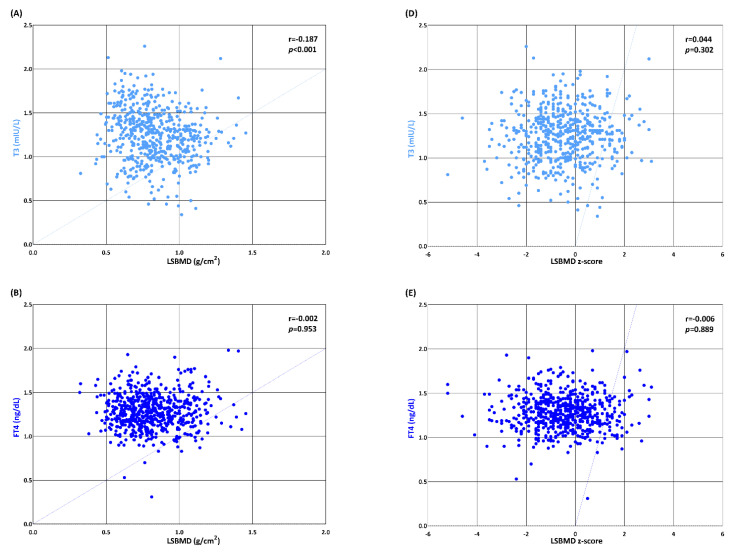

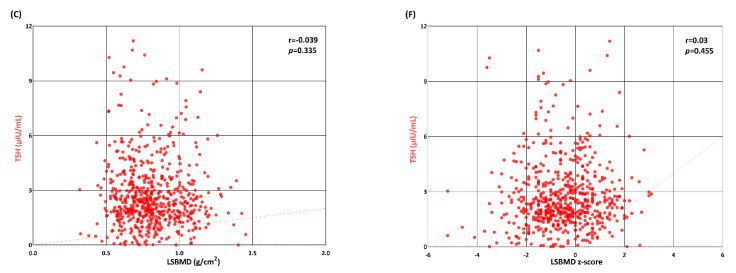
Scatter plot representing the correlations of lumbar spine bone mineral density (LSBMD) (**A**–**C**) and its z-scores (**D**–**F**) and with triiodothyronine (T3), free thyroxine (FT4), and thyrotropin (TSH).

Both the areal BMD (g/cm^2^) and z-scores for the lumbar spine showed no direct correlation with serum FT4 or TSH levels (Figure 3). The serum T3 level was not correlated with LSBMD z-scores but was negatively correlated (r = 0.044, *p* = 0.302) with LSBMD (g/cm^2^). In the univariate regression, the height (β = 0.42, *p* < 0.001), weight (β = 0.41, *p* < 0.001), and BMI (β = 0.31, *p* < 0.001) z-scores as well as the RFBMD (β = 0.73, *p* < 0.001) and LFBMD (β = 0.74, *p* < 0.001) z-scores were significantly associated with the LSBMD z-scores, while the serum T3, FT4, and TSH levels were not (Table 3). The BMI z-score (β = 0.32, *p* < 0.001) was independently related to the LSBMD z-scores, while serum T3, FT4, and TSH levels showed no association with the LSBMD z-scores in the following multivariate regression analyses.

In a subsequent multiple logistic regression analysis, the unadjusted ORs of abnormal TSH levels for LSBMD z-scores of less than −1.0, −2.0, and −3.0 were 1.85 (95% CI = 1.08–3.17; *p* = 0.026), 2.4 (95% CI = 1.23–4.7; *p* = 0.011), and 3.89 (95% CI = 1.34–11.34; *p* = 0.0.013), respectively (Table 4). The unadjusted-abnormal T3 level had an OR of 2.38 (95 % CI = 1.07–5.25; *p* = 0.033) for an LSBMD z-score of less than −2.0, but it was not significant for LSBMD z-scores of less than −1.0 and −3.0. Unadjusted abnormal FT4 levels were not associated with the LSBMD z-scores at any time interval. After adjusting for age, sex, BMI, and underlying conditions, the ORs of abnormal TSH levels were greater within all intervals of LSBMD z-scores and increased as the LSBMD z-scores decreased (OR = 2.25, 95% CI = 1.24–4.08, *p* = 0.008 for LSBMD z-score less than −1.0; OR = 2.65, 95% CI = 1.29–5.46, *p* = 0.008 for less than −2.0; OR = 6.21, 95% CI = 1.8–21.41, *p* = 0.004 for less than −3.0). Even after adjusting for confounders, the abnormal T3 and FT4 levels were still not significantly associated with decreased LSBMD z-scores.

## 4. Discussion

Although the direct causality between abnormal thyroid function and low bone mass remains controversial, an abnormality in TSH level seems to exert a more sensitive effect on pediatric bone fragility compared with TH. Owing to the variety of underlying systemic conditions and the fact that their treatment-associated factors simultaneously result in severe secondary osteoporosis and thyroid illness, recognizing which is more affected is as important as identifying the causes. The association between thyroid function and bone assessment in children and adolescents with a wide range of chronic illnesses suggests that abnormal TSH levels, whether elevated or decreased, could become a susceptibility indicator closely linked to decreased BMD; thus, it could be considered an early predictor of secondary osteoporosis. This information may prompt any pediatric healthcare provider, including pediatric endocrinologists, to closely monitor the thyroid function with skeletal assessment to prevent further complications, such as fracture.

The recently developed evidence-based consensus guidelines and recommendations for therapeutic intervention and prevention of secondary osteoporosis have pointed out that the hyperthyroidism-induced acceleration of bone turnover followed by increased cartilage maturation and bone resorption is a vital mechanism of bone loss [8,9,17]. Recent guidelines published by the Korean Thyroid Association highlighted that bone health assessment is critical in adult patients with endogenous overt hyperthyroidism or hyperthyroidism induced by TSH suppression therapy for differentiated thyroid cancer [18]. On the contrary, hypothyroidism might slow the rate of bone resorption; however, its existence could hinder the activity of osteoblasts, leading to impaired bone formation and turnover, which is critical, especially in a growing child [19]. Multiple chronic childhood illnesses requiring aggressive and lifelong care with a combination of different treatment regimens can affect the intact hypothalamic–pituitary–thyroid (HPT) axis, leading to thyroid dysfunction. For example, glucocorticoids, the most widely used drugs for controlling the inflammatory and autoimmune reactions, not only suppress the secretion of thyrotropin-releasing hormones (TRHs) and TSHs by altering the HPT axis, but also diminish the TH secretion by reducing the deiondinase activity [20]. Whether excessive or deficient TH level can adversely affect bone loss remains unknown; hence, maintaining euthyroidism prevents further progression to secondary osteoporosis during childhood.

TSH secretion is stimulated by thyrotrophs in the anterior pituitary gland and is mainly regulated by TRH release driven by the hypothalamic THR neurons. TSH signals the thyroid follicular cells to produce T3 and T4, whose excess exerts an inhibitory action via a negative feedback loop system on the anterior pituitary gland, thus suppressing the TSH secretion. TSH receptor expression was observed not only in the thyroid tissue but also in normal osteoblasts, implying the direct action of TSH on bone formation [21]. Several observational studies conducted in an adult population concluded that individuals with low normal TSH levels, regardless of TH levels, are at risk of developing osteoporosis [22]. However, BMD loss was more likely attributed to the diverse immune–skeletal–endocrine interactions between TSH in the bone marrow cells and TSH receptors in the skeletal tissues based on the osteoclastogenic effect of tumor necrosis factor α and the expression of splice variants of TSH [23].

Upon the TSH surge occurring at birth, the elevated TSH level begins to gradually reduce and plateau between 6 and 12 months of age; meanwhile, the surge could be somewhat attenuated or prolonged in prematurely born infants. The age-specific reference intervals for TSH in pediatric populations are much wider than those in adults, with upper limits ranging up to 39.0, 12.5, and 8.0 mU/L at 7 days, 3 months, and 5–10 years of age, respectively [16,24]. Without the classic symptoms of hypothyroidism, TSH screening is commonly performed in pediatric endocrinology clinics, because the elevation of TSH levels is frequently observed in association with childhood obesity, short stature, and delayed or accelerated pubertal progression [25]. Low TSH levels mostly occur in patients with TH-excessive conditions; however, the level of TSH seems to decrease, specifically in children diagnosed with eating disorders with abrupt weight loss or in patients who are taking high-dose glucocorticoids, dopamine, and amiodarone [26,27]. Due to its finely tuned and sensitively controlled negative feedback loop, the regulation of TSH secretion can easily be affected in children with chronic illnesses, which is currently referred to as non-thyroidal illness syndrome and commonly manifests as subclinical hypothyroidism [28]. Majority of our study participants from five different disease categories had normal TSH, T3, and FT4 levels at DXA assessment; meanwhile, the proportions of patients with abnormal TSH, T3, and FT4 levels were 9.5%, 7.4%, and 0.5%, respectively. The effect of pediatric chronic diseases on HPT axis dysfunction through different mechanisms seems to result in TSH alteration; however, insufficient TH levels may trigger overt hyperthyroidism or hypothyroidism of central origin. Since an abnormal TSH level is considered a potential parameter for predicting low LSBMD z-scores, the serum TSH levels should not be overlooked, despite having a normal TH status. Knowing the age-related reference interval may be critical in interpreting the association between thyroid function and bone health.

According to Sheng et al., TSH showed no significant correlation with BMD of euthyroid adults and was also not associated with the variations of BMD and the fracture risk in the elderly [29]. A similar study by Lin et al. yielded consistent results and suggested thyroid hormone, particularly T4, as a more appropriate indicator between bone and thyroid function, demonstrating weak negative correlations between T4 and BMD [30]. Both studies were conducted based on an adult population in euthyroid and disease-free states. Veldscholte et al. demonstrated no significant association between TSH and BMD (g/cm^2^) and BMC (g) of the total body without the head in a healthy 6-year-old population, and they also suggested that FT4 is a better indicator of bone turnover [13]. According to our results, based on the thyroid function of unhealthy patients and sex- and age-matched LSBMD z-scores, TSH showed no direct correlation with BMD as well. Taken together, it is agreed that there hardly exists a simple correlation between TSH and BMD, and BMD does not simply increase as the TSH increases or decreases. On the other hand, our subjects were a pediatric population of euthyroid state but having been diagnosed with various chronic illnesses affecting the HPT axis. There is not enough evidence to discuss the difference between adult and pediatric populations regarding the effect of bone mineral accrual on the regulation of the HPT axis. Nevertheless, the novelty of this study showed that any abnormal TSH level out of the age-specific reference interval, whether elevated or decreased, could be a sign of low LSBMD z-scores, and the link between these two factors became more evident in severely osteoporotic children. Compared with the OR of unadjusted-abnormal TSH for an LSBMD z-score of less than −1.0, a six-fold increase was observed in the abnormal TSH level for an LSBMD z-score of less than −3.0 when the abnormal TSH level was adjusted for age, sex, BMI, and underlying conditions. In addition, our results showed that FT4 levels could not be an indicator of low BMD. After all, it might be a unique characteristic occurring during childhood and adolescent periods.

Our study has several limitations. First, this was an age-unmatched, single-armed, cross-sectional study based on an unevenly distributed number of patients from each underlying disease category. Hemato-oncologic and rheumatoid diseases accounted for the majority (89.9%) of the total study participants. Second, children with abnormal TH and TSH levels comprised less than 10% of the total study participants, whereas one-third had relatively low bone mass with an LSBMD z-score of less than −1.0. Additionally, the number of participants with hyperthyroidism and triiodothyroninemia was relatively small to determine the effect of elevated T3 and T4 levels on bone mass. Third, the BMD z-scores of the lumbar spine were used to explore their causal relationship with thyroid function. Although the overall femoral BMD z-scores were lower than the LSBMD z-scores, the lumbar spine and total body-less head were the internationally preferred skeletal sites for performing areal BMD measurements in pediatric patients [31]. Since the national reference values for BMD are provided, additional measurements of BMD z-scores for total body-less head could have been useful to elucidate the connection between bone and thyroid function [15]. Nevertheless, to the best of our knowledge, our study is the first to examine the connection between thyroid function and BMD in children with various underlying conditions, and it aimed to identify which among the three thyroid function parameters more sensitively reflected a low BMD.

## 5. Conclusions

In conclusion, abnormal TSH levels showed a stronger association with low LSBMD z-scores compared with T3, FT4, and TSH levels. This parameter could be an early indicator of low BMD in children and adolescents with various underlying conditions. Therefore, immediate bone health assessment is recommended when the TSH levels, whether elevated or decreased, are outside the age-specific reference range. Larger prospective studies are warranted to further examine the role of TSH in bone fragility in order to prevent pediatric osteoporosis.

## Figures and Tables

**Table 1:** Clinical description of the study subjects.

	Total (*n* = 619)
**Male, *n* (%)**	312 (50.9)
**Age, y**	13.22 ± 3.23
**Anthropometry, z-score**	
Height	−0.51 ± 1.27
Weight	−0.22 ± 1.48
Body mass index	0.03 ± 1.52
**Underlying condition, *n* (%)**	
Hemato-oncologic	493 (79.6)
Rheumatoid	64 (10.3)
Gastrointestinal	23 (3.7)
Endocrinologic	19 (3.1)
Miscellaneous	20 (3.2)
**Age at diagnosis, years**	9.26 ± 11.86
**Bone mineral density**	
Lumbar spine, g/cm^2^	0.81 ± 0.19
Lumbar spine, z-score	0.49 ± 1.28
Low bone mass, *n* (%)	
≤0	432 (70.5)
≤−1.0	231 (37.7)
≤−2.0	72 (11.7)
≤−3.0	18 (2.9)
Right femur, g/cm^2^	0.76 ± 0.16
Right femur, z-score	−1.04 ± 1.32
Left femur, g/cm^2^	0.77 ± 0.16
Left femur, z-score	−1.03 ± 1.34
**Thyroid function**	
T3, ng/mL	1.25 ± 0.29
Interpretation, *n* (%)	
Normal	505 (92.7)
Low	39 (7.2)
High	1 (0.2)
FT4, ng/dL	1.28 ± 0.19
Interpretation, *n* (%)	
Normal	614 (99.5)
Low	3 (0.5)
High	0 (0)
TSH, µU/mL	2.76 ± 1.87
Interpretation, *n* (%)	
Normal	560 (90.5)
Low	36 (5.8)
High	23 (3.7)

All values are expressed as mean ± standard deviation unless mentioned. FT4, free thyroxine; T3, triiodothyronine; TSH, thyrotropin.

**Table 2:** The comparison of bone mineral density z-scores when triiodothyronine, free thyroxine, and thyrotropin levels were normal and abnormal.

z-Scores	T3			FT4			TSH		
	Normal	Abnormal	*p*	Normal	Abnormal	*p*	Normal	Abnormal	*p*
**LSBMD**	−0.47 ± 1.26	−0.67 ± 1.4	0.357	−0.48 ± 1.28	−1.23 ± 1.53	*0.31*	**−0.45 ± 1.25**	**−0.86** **±** **1.46**	***0.018***
**RFBMD**	−1.03 ± 1.28	−1.17 ± 1.7	0.537	−1.04 ± 1.33	−0.8 ± 1.4	*0.75*	**−1.01 ± 1.32**	**−1.39** **±** **1.35**	***0.036***
**LFBMD**	−1.01 ± 1.3	−1.31 ± 1.73	0.211	−1.03 ± 1.34	−1.0 ± 1.56	*0.969*	**−0.98 ± 1.32**	**−1.49** **±** **1.48**	***0.007***

All values are expressed as mean ± standard deviation. FT4, free thyroxine; LFBMD, left femur bone mineral density; LSBMD, lumbar spine bone mineral density; RFBMD, right femur bone mineral density; T3, triiodothyronine; TSH, thyrotropin.

**Table 3:** Univariate and multivariate regression analyses of factors associated with the lumbar spine bone mineral density ^†^.

Clinical Parameter	Univariate			Multivariate		
	β (95% CI)	SE	*p*	β (95% CI)	SE	*p*
**Underlying condition**						
** Hemato-oncologic**	−0.15 (−0.18–0.49)	0.17	*0.371*	0.18 (−0.15–0.51)	0.17	*0.291*
** Rheumatoid**	−0.27 (−0.81–0.27)	0.27	*0.324*	0.27 (−0.31–0.86)	0.29	*0.359*
** Gastrointestinal**	−0.22 (−0.81–0.36)	0.29	*0.455*	0.13 (−0.57–0.84)	0.36	*0.709*
** Endocrinologic**	0.04 (−0.54–0.61)	0.29	*0.9*	0.29 (−0.29–0.87)	0.29	*0.322*
**^†^ Height**	**0.42 (0.35–0.49)**	**0.04**	***<0.001***			
**^†^ Weight**	**0.41 (0.35–0.47)**	**0.03**	***<0.001***			
**^†^ BMI**	**0.31 (0.25–0.37)**	**0.03**	***<0.001***	**0.32 (0.26–0.39)**	**0.03**	***<0.001***
**^†^ RFBMD**	**0.73 (0.68–0.78)**	**0.03**	***<0.001***			
**^†^ LFBMD**	**0.74 (0.69–0.79)**	**0.03**	***<0.001***			
**T3**	0.19 (−0.17–0.55)	0.18	*0.302*	−0.06 (−0.41–0.29)	0.18	*0.749*
**FT4**	−0.04 (−0.55–0.48)	0.26	*0.889*	−0.05 (−0.44–0.55)	0.25	*0.833*
**TSH**	0.02 (−0.03–0.07)	0.03	*0.455*	0.01 (−0.04–0.07)	0.03	*0.637*

**^†^** Indicates sex- and age-matched z-scores. BMI, body mass index; CI, confidence interval; FT4, free thyroxine; LFBMD, left femur spine bone mineral density; RFBMD, right femur bone mineral density; T3, triiodothyronine; TSH, thyrotropin; SE, standard error.

**Table 4:** Logistic regression analyses of abnormal triiodothyronine, free thyroxine, and thyrotropin when using decreased lumbar spine bone mineral density z-scores as dependent variables.

Clinical Parameter	LSBMD z-Score ≤ −1.0			LSBMD z-Score ≤ −2.0			LSBMD z-Score ≤ −3.0		
	OR (95% CI)	SE	*p*	OR (95% CI)	SE	*p*	OR (95% CI)	SE	*p*
**Abnormal T3**									
** Unadjusted**	1.02 (0.53–1.98)	0.34	*0.953*	**2.38 (1.07–5.25)**	**0.41**	***0.033***	0.97 (0.12–7.61)	1.05	*0.977*
** ^†^ Adjusted**	0.93 (0.43–2.01)	0.39	*0.931*	2.29 (0.92–5.69)	0.47	*0.075*	1.05 (0.11–10.36)	0.04	*0.968*
**Abnormal FT4**									
** Unadjusted**	3.39 (0.31–37.55)	1.23	*0.32*	3.82 (0.34–42.71)	1.23	*0.276*	<0.01 (<0.01–0.01)	1385.38	*0.992*
** ^†^ Adjusted**	1.81 (0.14–23.24)	1.3	*0.649*	2.43 (0.18–32.13)	1.32	*0.499*	<0.01 (<0.01–0.01)	<0.01	*0.996*
**Abnormal TSH**									
** Unadjusted**	**1.85 (1.08–3.17)**	**0.27**	***0.026***	**2.4 (1.23–4.7)**	**0.34**	***0.011***	**3.89 (1.34–11.34)**	**0.55**	***0.013***
** ^†^ Adjusted**	**2.25 (1.24–4.08)**	**0.3**	***0.008***	**2.65 (1.29–5.46)**	**0.37**	***0.008***	**6.21 (1.8–21.41)**	**2.89**	***0.004***

**^†^** T3, FT4, and TSH were adjusted for age, sex, body mass index, and underlying condition. CI, confidence interval; FT4, free thyroxine; LSBMD, lumbar spine bone mineral density; OR, odds ratio; T3, triiodothyronine; TSH, thyrotropin; SE, standard error.

## Data Availability

The data presented in this study are available on request from the corresponding author. The data are not publicly available due to the privacy of research participants.
